# SRCIN1 Regulated by circCCDC66/miR-211 Is Upregulated and Promotes Cell Proliferation in Non-Small-Cell Lung Cancer

**DOI:** 10.1155/2020/5307641

**Published:** 2020-09-09

**Authors:** Weijun Hong, Suyun Yu, Yaqing Zhuang, Qingqing Zhang, Jiqin Wang, Xiwen Gao

**Affiliations:** ^1^Department of Respiratory Medicine, Minhang Hospital, Fudan University, China; ^2^Department of Emergency Medicine, Minhang Hospital, Fudan University, China

## Abstract

The incidence and mortality of lung cancer were extremely high. The present study showed that SRCIN1 was an oncogene in non-small-cell lung cancer (NSCLC). Public dataset analysis showed SRCIN1 was significantly overexpressed in NSCLC samples. Also, we found that NSCLC patients with higher SRCIN1 expression had shorter OS time by analyzing TCGA, Kaplan-Meier Plotter, GSE30219, GSE50081, and GSE19188 databases. Overexpression or knockdown of SRCIN1 significantly induced or reduced A549 and H1299 cell proliferation. Furthermore, we found SRCIN1 was directly targeted by miR-211. Overexpression or knockdown of miR-211 suppressed or induced SRCIN1 levels in NSCLC. Moreover, we found that miR-211 affected NSCLC cell proliferation through SRCIN1. Previous studies demonstrated that circRNAs could act as miRNA sponges in cancer cells. In this study, we showed that knockdown of circCCDC66 induced expression of miR-211. Luciferase assay demonstrated that miR-211 suppressed the activity of luciferase reporter-contained circCCDC66 sequences. Moreover, knockdown of circCCDC66 significantly inhibited SRCIN1 levels in both A549 and H1299 cells. These results showed that circCCDC66 acted as a miRNA sponge to affect the miR-211/SRCIN1 axis. Of note, we for the first time revealed that circCCDC66 suppression reduced cell proliferation by about 65% in A549 and by about 40% in H1299 cells. We thought this study could provide novel potential biomarkers for NSCLC.

## 1. Background

As global cancer statistics showed in 2018, the incidence and mortality of lung cancer were extremely higher compared to other tumors [1]. Lung cancer is classified into NSCLC (non-small-cell lung cancer) and SCLC (small-cell lung cancer) [2]. Most lung cancer patients belong to NSCLC, which includes squamous cell carcinoma, adenocarcinoma, and large carcinoma [3]. Although medical imaging is a largely used strategy to screen lung cancer, an increasing number of NSCLCs can be diagnosed at the initial phase, and the case fatality rate for NSCLC is still high [4]. Due to the massive population base, the increasing number of smokers, and high health care costs in some countries, a majority of people are diagnosed with late-stage disease [5, 6]. Therefore, although many treatment approaches have been developed for NSCLC, the therapeutic outcomes are not optimal [7]. Therefore, determining the underlying role of NSCLC and further uncovering the unknown indicator are really of importance, followed by ameliorating early diagnosis and providing promising treatment for NSCLC patients.

SRC Kinase Signaling Inhibitor 1 (SRCIN1) acts as a regulator for inhibiting cell spreading and migration and is also involved in calcium-dependent exocytosis. SRCIN1 is identified to be an inhibitor in lung cancer and breast cancer along with osteosarcoma [8]. For instance, Wang et al. showed that increased expression of SRCIN1 could result in reduced cell growth of osteosarcoma via promoting E-cadherin expression in vitro [9]. Chen et al. demonstrated that enhanced SRCIN1 repressed proliferation of human liver cancer cells and blocked epithelial-mesenchymal transition in cell line HepG2 [10]. Although some researches have been carried out on SRCIN1, there is a very little scientific understanding of the roles of SRCIN1 in the development of tumors. It is therefore necessary to study the roles and functions of SRCIN1 in tumorigenesis and progression of NSCLC.

miRNAs are small noncoding RNAs containing less than 30 nucleotides. miRNAs are crucial posttranscriptional regulators in cells. Mature miRNAs target mRNA transcripts through complementary base-pairing to the 3′UTR, thus resulting in target mRNA degradation or translational inhibition. In this way, miRNAs act as a regulator widespread in cancer cells [11]. Therefore, changes in miRNA expression or miRNA imbalance may affect the cell cycle progression, which in turn affects the fate and behavior of tumor cells [12]. Previous research has shown that miRNAs played a carcinogenic or suppressive role in the progression of NSCLC. For example, highly expressed miR-10a would induce cell proliferation migration and invasion of NSCLC by targeting PTEN [13]. Jiang et al. revealed that miR-17, miR-20a, and miR-20b were regarded as inhibitors of TGF-beta receptor 2, thus rescuing cisplatin-resistant and retarding metastasis of NSCLC [14]. TRAIL, a TNF-related apoptosis-inducing ligand, is a novel molecular against tumors because it has the ability of selective inhibition of apoptosis in tumors in the absence of side effects on nearby normal cells [15]. The TRAIL-mediated suppressed tumor route is reduced in numerous tumors containing lung cancer [16]. Iaboni et al. demonstrated that overexpression of the tumor suppressor miR-212 could restore the effectiveness of TRAIL treatment by inhibiting PED/PEA-15 (antiapoptotic protein) in NSCLC cells [17]. The abovementioned studies showed that miRNAs played a key role in the progression and development of NSCLC. Therefore, it is important to explore molecular mechanism aspects to better understand NSCLC pathogenesis.

circRNA in eukaryotes was discovered in the 1970s by transmission electron microscopy [18]. However, we know very little about the structures and functions of circRNA before the maturity of high-throughput sequencing and bioinformatics. Numerous studies have found that circRNAs are abundant and have many functions in eukaryotic cells, including regulating parental gene level and functioning as microRNA (miRNA) sponges. Besides, some studies have demonstrated that circRNAs participated in the growth of cancer [19]. For example, Li et al. found that the hsa_circ_002059 level was obviously linked to distal metastasis, TNM stage, gender, and age in GC (gastric cancer) patients [20]. Yao et al. showed that higher expression of hsa_circ_100876 was positively related to the metastasis of the lymph node and carcinoma stage in NSCLC [21]. Zhong et al. indicated that the expression of circRNA-MYLK and circTCF25 was evidently enhanced in bladder cancer tissues [22]. All the data indicated that the abnormal expression of circRNAs could act as a newly produced indicator for tumor development.

Competitive endogenous RNA (ceRNA) regulation suggested a complex network of transcriptional RNAs including long noncoding RNAs (lncRNAs) and circRNA, which can act as natural miRNA sponges to inhibit miRNA functions and modulate mRNA expression [23, 24]. Here, we attempted to investigate the functions and mechanisms of circCCDC66, SRCIN1, and miR-211 in NSCLC. Our results showed that decreased SRCIN1 could inhibit cell proliferation, migration, and invasion. Furthermore, luciferase assay showed that SRCIN1 was a direct target of miR-211, which was also sponged by circCCDC66. Collectively, our findings suggested several novel biomarkers for NSCLC.

## 2. Methods and Materials

### 2.1. Tissues

Twenty NSCLC tissues and twenty normal ones on average 5 cm from the indicated tumor of patients which was surgically removed were provided by Minhang Hospital, Fudan University. Among them, none were subjected to radiation and chemotherapy beforehand. All the experiments were approved by the corresponding Ethics Committee and unanimous consent by all subjects with signed informed documents. The tissues in this study were quickly put into liquid nitrogen after dissecting from patients, followed by preserving them in -80°C for long-term use.

### 2.2. Cells

H1299 and A549 (human NSCLC cells) were purchased from the ATCC (Manassas, USA) and then cultured in RPMI 1640 medium (Gibco, USA) containing 10% FBS (Gibco) and 1% penicillin/streptomycin under 37°C incubator with 5% CO_2_.

### 2.3. qRT-PCR (Quantitative Real-Time Reverse Transcription PCR)

Trizol (TaKaLa, China) was used to extract total RNA from cells and tissues as described in the manual. Reverse transcription system was performed as follows: 10 *μ*L volume included 500 ng RNA with Prime Script RT Master Mix (RiboBio, China) and RNAase free ddH_2_O, followed by being subjected to qRT-PCR with indicated primers by a SYBR Master Mix (ABI, USA) on an ABI 7500 system (ABI). We finally calculated relative RNA expression by the 2^−*ΔΔ*Ct^ method.

### 2.4. Plasmid Construction and Transfection

All the oligonucleotides were synthesized by GenePharma (Shanghai, China). The designed siRNA targeting circCCDC66 (si-circCCDC66) was for the covalently closed junction. PcDNA3.1 vector expressing si-circCCDC66 (5′-GAGCAUCAGGAAACAGUAC-3′) was constructed to ablate the circCCDC66 level. 50 pmol/mL of si-circCCDC66 and negative control (NC) inhibitor was separately transfected into indicated cells by Lipofectamine 2000 (Invitrogen) as per the manual's instructions, followed by changing the medium at 6 hours posttransfection.

### 2.5. CCK-8 Assay

Cell proliferation assay was conducted by a Cell Counting Kit (Dojindo, Japan) at 48 hours posttransfection. 1 × 10^4^ cells of H1299 and A549 per well were reseeded in 96 wells and then 10 *μ*L of CCK-8 solution was added at the indicated time for 2 hours of incubation. Cell proliferation was measured at specified days. Absorbance value in 450 nm was detected by an Infinite M200 plate reader (Tecan, Switzerland).

### 2.6. Transwell Assay

8 *μ*m pores of a transwell chamber (Costar, USA) and Matrigel film (BD Biosciences) used for coating upper chambers were successively applied to conduct an invasion assay. The treatment chamber including 100 *μ*L of medium absence serum and the lower chamber with 600 *μ*L of medium containing 5% FBS were seeded with1 × 10^4^ cells as indicated at 37°C with 5% CO_2_ overnight. On the following day, nonimmigrated or noninvasive cells on the top side were eliminated by cotton swab. The insert was fixed by methanol for 20 minutes and then dyed by DAPI at concentration of 10 *μ*g/mL for 5 minutes. We counted migrated or invade cells to the membrane bottom and captured images by microscope from three independent experiments in triplicate.

### 2.7. Luciferase Assay

The circCCDC66 fragment containing mutated (mut) or wild-type (wt) seed region was inserted into the psiCHECK-2 construct (ABI). Wt or mut circCCDC66, miR-211 mimic, or mimic control was separately transfected into 1 × 10^5^ cells per well of A549 and H1299 by Lipofectamine 2000 (Invitrogen). Relative luciferase activity was detected by a dual-luciferase reporter kit at 48-hour induction (Promega, USA). WT 3′UTR of SRCIN1 mRNA with the assumed binding site of miR-211 was ligated downstream of the firefly luciferase expression cassette in the pMIR-REPORT vector (Thermo Scientific). The positive clones were named by pMIR-SRCIN1-3′UTR (SRCIN1-wt). The pMIR-SRCIN1-3′UTR-mut (SRCIN1-mut) plasmid was generated by the QuikChange Mutagenesis kit (Stratagene, USA) which referred to the abovementioned. SRCIN1-wt or SRCIN1-mut with miR-211 mimics or NC mimics was then transfected into A549 and H1299 cells by Lipofectamine 2000.

### 2.8. Statistical Analysis

The representative data are shown as mean ± SD. All the data was calculated after three independent experiments, followed by the limma package. The value of gene expression conformed to the normal distribution. The difference existing in two comparison groups or multiple groups in the indicated experiments was determined by Student's *t*-test. The differences between tumor and normal tissues were counted by paired-sample *t*-test. The linear relationship occurring in either two groups in NSCLC tissues was detected by the Pearson correlation coefficient. The obvious difference was indicated as *P* value less than 0.05.

## 3. Results

### 3.1. SRCIN1 Was Upregulated and Correlated to Shorter Survival Time in NSCLC

**Figure 1:**
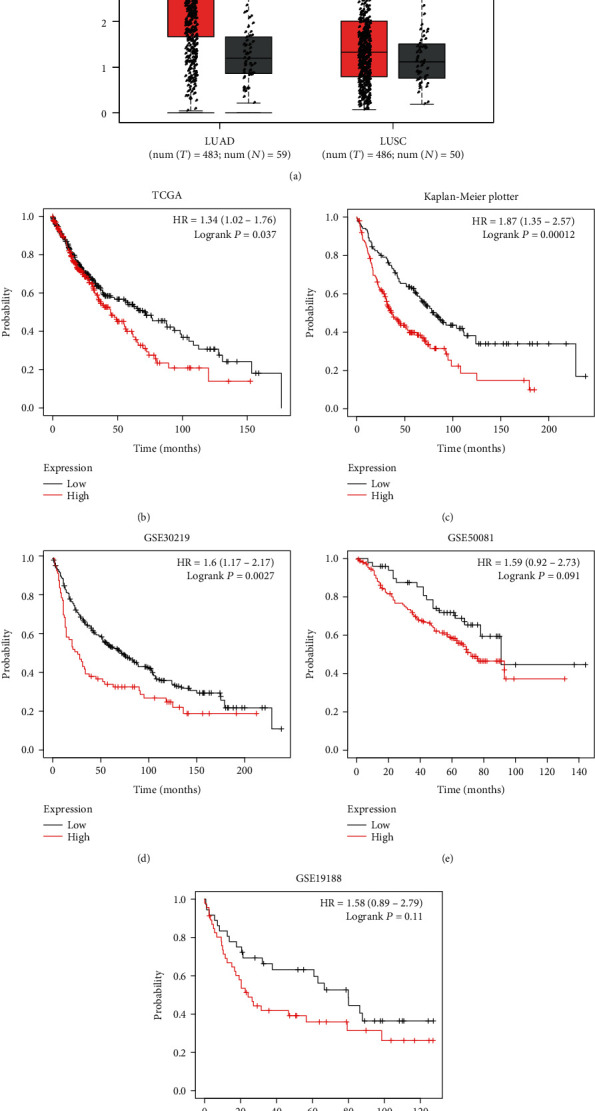
SRCIN1 was upregulated and correlated to shorter survival time in NSCLC. (a) SRCIN1 was upregulated in both lung adenocarcinoma and lung squamous cell carcinoma samples. (b) NSCLC patients with higher SRCIN1 expression had shorter overall survival (OS) time. (c–f) Overexpression of SRCIN1 was related to shorter OS time in NSCLC by analyzing the Kaplan-Meier Plotter (c), GSE30219 (d), GSE50081 (e), and GSE19188 databases (f). ^∗^*P* < 0.05.

As presented in Figure 1(a), we observed that SRCIN1 was upregulated in both lung adenocarcinoma and lung squamous cell carcinoma samples compared to that in match normal tissues, suggesting that SRCIN1 was related to the tumorigenesis of NSCLC.

Also, we calculated the association between SRCIN1 levels and overall survival (OS) time by using TCGA data (Figure 1(b)). We found that NSCLC patients with higher SRCIN1 expression had shorter OS time. To further confirm this, we analyzed microarray data related to NSCLC. We also observed that overexpression of SRCIN1 was related to shorter OS time in NSCLC by analyzing the Kaplan-Meier Plotter (Figure 1(c)), GSE30219 (Figure 1(d)), GSE50081 (Figure 1(e)), and GSE19188 databases (Figure 1(f)).

### 3.2. SRCIN1 Acted as an Oncogene in NSCLC Cells

**Figure 2:**
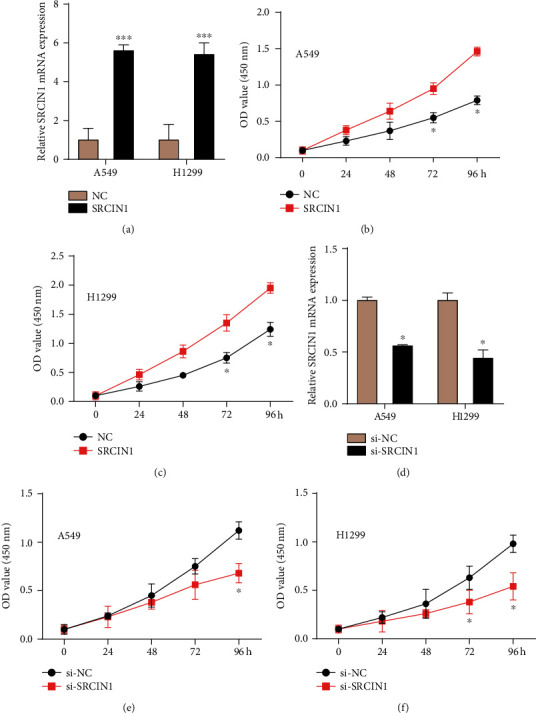
SRCIN1 acted as an oncogene in NSCLC cells. (a) Upregulated SRCIN1 could result in a higher level of SRCIN1. (b, c) Overexpressed SRCIN1 could induce A549 and H1299 cell growth. (e, f) Knockdown of SRCIN1 suppressed cell proliferation in A549 (e) and H1299 (f). ^∗^*P* < 0.05, ^∗∗∗^*P* < 0.001.

We next validated the SRCIN1 role involved in A549 and H1299 cell metastases. Our findings revealed that upregulated SRCIN1 could result in a higher level of SRCIN1 in both A549 and H1299 cells (Figure 2(a)). Then, we aimed to assess the influences of SRCIN1 induced on cell proliferation. The CCK-8 assay was applied and showed that SRCIN1 would induce A549 and H1299 cell growth (Figures 2(b) and 2(c)). However, knockdown of SRCIN1 suppressed cell proliferation in A549 and H1299 (Figures 2(e) and 2(f)). These results showed that SRCIN1 was an oncogene in NSCLC.

### 3.3. SRCIN1 Was a Target of miR-211 in NSCLC Cells

**Figure 3:**
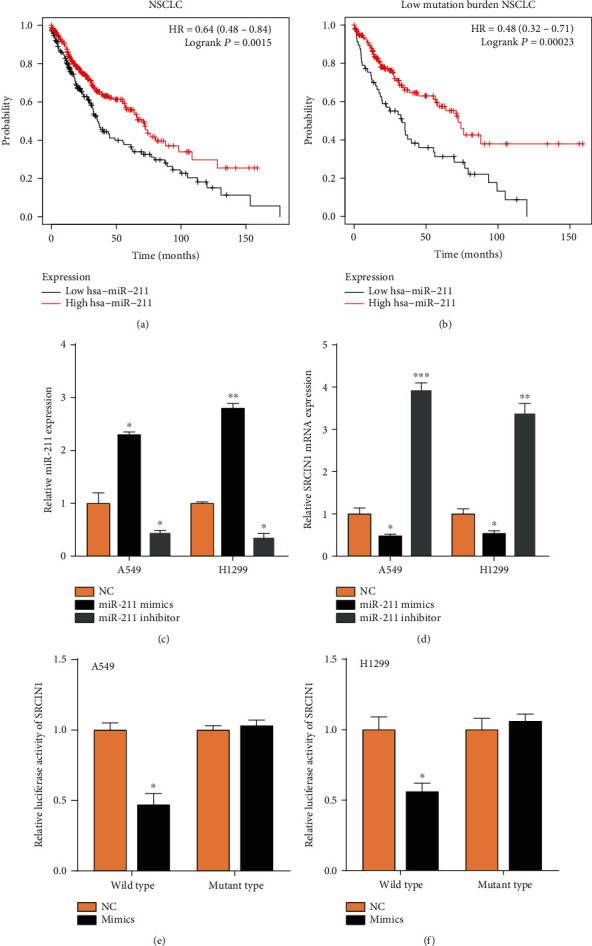
SRCIN1 was a target of miR-211 in NSCLC cells. (a, b) Higher expression of miR-211 was related to longer OS time in NSCLC, especially in low mutation-burden NSCLC. (c) Effects of transfected miRNA mimics or inhibitors on miR-211 levels in cells. (d) SRCIN1 was suppressed by the miR-211 mimics and induced by the inhibitors. (e, f) The cells cotransfected with miR-211 mimics and wild-type SRCIN1 showed lower luciferase activity. ^∗^*P* < 0.05, ^∗∗^*P* < 0.01, ^∗∗∗^*P* < 0.001.

Here, we wanted to predict and investigate the upstream miRNAs of SRCIN1 by bioinformatics. For the following studies, we selected miR-211 as the candidate miRNA that targeted SRCIN1. miR-211 was reported as a tumor suppressor in multiple cancers. Higher expression of miR-211 was related to longer OS time in NSCLC, especially in low mutation-burden NSCLC (Figures 3(a) and 3(b)), suggesting that miR-211 might be a tumor suppressor.

**Figure 4:**
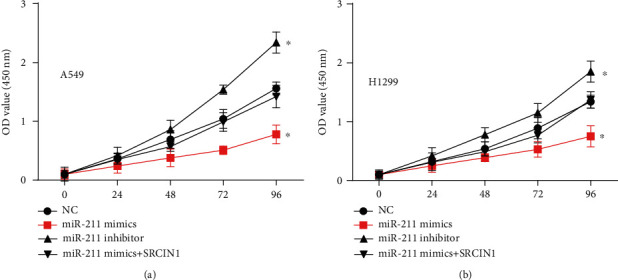
The effect of miR-211/SRCIN1 on cell growth in NSCLC cells. (a, b) In A549 and H1299, the upregulation of miR-211 inhibited the proliferation rate, while the downregulation of miR-211 increased the proliferation rate, and the overexpression of SRCIN1 weakened the inhibitory effect of miR-211 overexpression on cell proliferation. ^∗^*P* < 0.05.

Then, we transfected miRNA mimics or inhibitors to modulate miR-211 (*P* < 0.05, Figure 3(c)). qRT-PCR showed that SRCIN1 was suppressed by the miR-211 and induced by the inhibitors (Figure 3(d)). Of note, a dual-luciferase reporter assay confirmed that SRCIN1 was a direct target of miR-211 (Figures 3(e) and 3(f)). Furthermore, we detected the effect of miR-211/SRCIN1 on cell growth in NSCLC cells (Figures 4(a) and 4(b)). Our results showed that miR-211 upregulation suppressed the A549 and H1299 proliferation rates, but miR-211 downregulation enhanced the A549 and H1299 proliferation rates. Moreover, we found that SRCIN1 overexpression recused the suppressive effect of miR-211 overexpression on cell proliferation in both A549 and H1299.

### 3.4. circCCDC66 Targeted miR-211 and Affected SRCIN1 Expression

**Figure 5:**
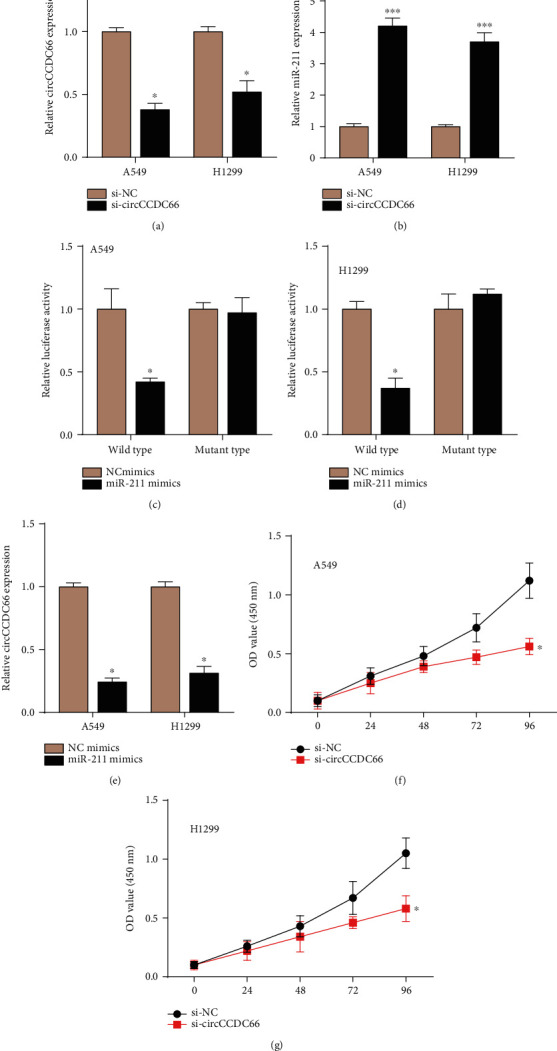
circCCDC66 targeted miR-211 and knockdown circCCDC66 suppresses cell proliferation in NSCLC. (a, b) Knockdown of circCCDC66 suppressed circCCDC66 levels and induced expression of miR-211. (c, d) miR-211 suppressed the luciferase activity of cells containing circCCDC66 sequences. (e) Overexpression of miR-211 in cells reduced circCCDC66 expression. (f, g) Inhibited circCCDC66 reduced cell proliferation. ^∗^*P* < 0.05, ^∗∗∗^*P* < 0.001.

Previous studies demonstrated that circRNAs could act as miRNA sponges in cancer cells. The prediction showed that circCCDC66 targeted miR-211. The qRT-PCR assay showed that the knockdown of circCCDC66 suppressed endogenous levels of this circRNA and induced the expression of miR-211 (Figures 5(a) and 5(b)). Luciferase assay demonstrated that miR-211 suppressed the activity of luciferase reporter-contained circCCDC66 sequences (Figures 5(c) and 5(d)). Moreover, knockdown of circCCDC66 significantly inhibited SRCIN1 levels in both A549 and H1299 cells.

### 3.5. Knockdown circCCDC66 Suppresses Cell Proliferation in NSCLC

We moved forward to identify the functionality of circCCDC66 in NSCLC. Our data revealed that inhibited circCCDC66 reduced cell proliferation by about 65% in A549 and by about 40% in H1299 cells (Figures 5(f) and 5(g)). This finding showed that circCCDC66 acted as an oncogenetic circRNA in NSCLC.

## 4. Discussion

In our study, we firstly determined SRCIN1 expression and molecular functions. The data revealed that SRCIN1 increased in NSCLC tissues compared to that in normal ones. Higher expression levels of SRCIN1 correlated to shorter OS time in NSCLC patients. Secondly, we aimed to explore the association between SRCIN1 and circCCDC66 and their mechanism of involvement in tumor development. For that purpose, we performed cell viability detection. Our data revealed that circCCDC66 knockdown and SRCIN1 silencing resulted in reduced abilities of NSCLC cell proliferation. The mechanism studies demonstrated that circCCDC66 sponged miR-211 to modulate SRCIN1 expression.

The molecular functions of SARCIN1 in cancer cells are controversial. According to previous reports, SARCIN1 could act as either an oncogene or a tumor suppressor. For example, Xu et al. revealed that SRCIN1 significantly inhibited gastric cancer cell viability, migration, and invasion [8]. Wang et al. demonstrated enhanced SRCIN1-repressed cell viability and colony formation with the invasion of osteosarcoma [9]. Conversely, other studies confirmed that SRCIN1 could serve an oncogenic role. For instance, Zhang et al. revealed that SRCIN1 increased the capacity of colorectal cancer cell viability, migration, and invasion by activating Wnt/*β*-catenin signaling routes [25]. The present study showed that SARCIN1 played as an oncogene in NSCLC. SARCIN1 was upregulated in NSCLC samples. Overexpression or knockdown of SRCIN1 significantly induced or reduced A549 and H1299 cell proliferation.

Emerging researches have discovered that miRNAs had an elementary function in carcinogenesis and NSCLC development [26–28]. For example, Gu et al. exhibited miR-940-mediated inhibition of the NSCLC cell proliferation by regulating its downstream target gene FAM83F [29]. Zhang et al. demonstrated that miRNA miR-21 promoted growth and invasion in NSCLC by controlling the expression of tumor suppressor PTEN [30]. Interestingly, Jiang et al. revealed that hsa-miR-125a-3p and hsa-miR-125a-5p had the same geographical origin but with the opposite function in NSCLC [31]. The former suppressed cell migration and invasion, while the latter enhanced tumor development. Our data suggested that the overexpression of miR-211 correlated to longer OS in NSCLC. Overexpression of miR-211 suppressed NSCLC cell viability. These functions indicate that miR-211 has potential as a new tumor marker for NSCLC.

The circRNA, a type of noncoding RNA, could extremely enrich microRNAs (miRNA) like a sponge [32]. They also could work on other kinds of RNAs by being base-paired [33]. In addition, circRNA could affect protein activity by binding with them [34]. Some researches show that circRNA can regulate the expression of host proteins, interact with RNA-binding proteins controlling transcription, play a role in transcriptional regulation in cis, and even regulate and control alternative splicing [35].

Our results showed that circCCDC66 promoted NSCLC cell proliferation in vitro. Besides, the concomitant knockdown of circCCDC66 led to a significant increase in miR-211 expression and a decrease in SRCIN1 expression. Many studies implied that circRNAs were involved in many biological processes competing for endogenous RNA (ceRNA) of miRNA. For example, circ_104075 which was identified as ceRNA increased the YAP level through absorbing miR-582-3p which in turn affected cell growth in hepatocellular carcinoma [36]. Cheng et al. reported that circTP63 functioning as the ceRNA of miR-873-3p promoted lung cell proliferation by decreasing the level of FOXM1 [37]. In this study, circCCDC66 regulated the progression of NSCLC by functioning as a ceRNA-like competitive adsorbent to absorb miR-211 and thus control SRCIN1 expression.

More experimental verification of our findings in NSCLC is needed. Our work revealed that SRCIN1 and circCCDC66 are oncogenic growth factors for NSCLC. Meanwhile, miR-211-caused tumor suppression has been found in NSCLC. Mechanically, we showed that circCCDC66 acted as a miRNA sponge to affect the miR-211/SRCIN1 axis. Our findings provided a new viewpoint into the posttranscriptional regulation mechanism of SRCIN1 and suggested that circCCDC66, SRCIN1, and miR-211 might act as a potential diagnostic biomarker for NSCLC. This regulatory mechanism also helps us to explore the occurrence and development of NSCLC from the perspective of transcriptional regulatory pairs and clearly understand the important role of circRNA (cricCCDC66) in this process. We will do our best to explore whether the regulatory axis plays a role in other cancers.

## Data Availability

The datasets used and/or analyzed during the current study are available from the corresponding author on reasonable request.
